# Huddling Conserves Energy, Decreases Core Body Temperature, but Increases Activity in Brandt's Voles (*Lasiopodomys brandtii*)

**DOI:** 10.3389/fphys.2018.00563

**Published:** 2018-05-18

**Authors:** Gansukh Sukhchuluun, Xue-Ying Zhang, Qing-Sheng Chi, De-Hua Wang

**Affiliations:** ^1^State Key Laboratory of Integrated Management of Pest Insects and Rodents, Institute of Zoology, Chinese Academy of Sciences, Beijing, China; ^2^University of Chinese Academy of Sciences, Beijing, China

**Keywords:** huddling, Brandt's voles, energetics, thermogenesis, core body temperature, activity

## Abstract

Huddling as social thermoregulatory behavior is commonly used by small mammals to reduce heat loss and energy expenditure in the cold. Our study aimed to determine the effect of huddling behavior on energy conservation, thermogenesis, core body temperature (T_b_) regulation and body composition in Brandt's voles (*Lasiopodomys brandtii*). Adult captive-bred female Brandt's voles (*n* = 124) (~50 g) in 31 cages with 4 individuals each were exposed to cool (23 ± 1°C) and cold (4 ± 1°C) ambient temperatures (T_a_) and were allowed to huddle or were physically separated. The cold huddling (Cold-H) groups significantly reduced food intake by 29% and saved digestible energy 156.99 kJ/day compared with cold separated groups (Cold-S); in cool huddling groups (Cool-H) the reduction in food intake was 26% and digestible energy was saved by 105.19 kJ/day in comparison to the separated groups (Cool-S). Resting metabolic rate (RMR) of huddling groups was 35.7 and 37.2% lower than in separated groups at cold and cool T_a_s, respectively. Maximum non-shivering thermogenesis (NSTmax) of huddling voles was not affected by T_a_, but in Cold-S voles it was significantly increased in comparison to Cool-S. Huddling groups decreased wet thermal conductance by 39% compared with separated groups in the cold, but not in the cool T_a_. Unexpectedly, huddling voles significantly decreased T_b_ by 0.25 – 0.50°C at each T_a_. Nevertheless, activity of Cold-H voles was higher than in Cold-S voles. Thus, huddling is energetically highly effective because of reduced metabolic rate, thermogenic capacity and relaxed T_b_ regulation despite the increase of activity. Therefore, Brandt's voles can remain active and maintain their body condition without increased energetic costs during cold exposure. This study highlights the ecological significance of huddling behavior for maintenance of individual fitness at low costs, and thus survival of population during severe winter in small mammals.

## Introduction

Winter is a stressful period for mammals when the majority ceases reproduction and allocates nutrients and fuel for the maintenance of the organism. Small mammals generally are more strongly affected than large mammals because energy requirements per unit of body mass are high due to the large surface area to volume ratio. Therefore, when environmental stressors persist for prolonged periods and available resources are limited small species will be challenged. To a large extent because of this, living in seasonal cold environment requires individual adjustments in morphology and physiology and also cooperative behavior by groups for communal nest sharing and storage of food (Wolff and Lidicker, 1981). Among cooperative behaviors huddling is an important social thermoregulatory behavior for group living species.

Huddling as an active and close aggregation of animals is used by many endotherms to reduce heat loss and lower energy expenditure and possibly allowing them to reallocate the saved energy to other functions such as growth or reproduction (Gilbert et al., 2010). Energetic advantages of huddling increase with lowered T_a_, increased group size and are mainly due to a reduced surface to volume ratio (Vickery and Millar, 1984; Canals et al., 1997; Nuñez-Villegas et al., 2014). The benefits of huddling in energy conservation (Putaala et al., 1995; Scantlebury et al., 2006; Kotze et al., 2008), local environment heating (Hayes et al., 1992; Nowack and Geiser, 2016) and survival (Sealander, 1952) have been studied in several species. In cold environments some small homeothermic mammals slightly lowered their core T_b_ (Chi and Wang, 2011; Nieminen et al., 2013). Yet huddling reduces the heat loss, its effect on core T_b_ regulation in huddling animals remains controversial (Andrews et al., 1987; Boix-Hinzen and Lovegrove, 1998; Fortin et al., 2000). Moreover, cold-exposed group of 4 mice exhibited a substantial increase in total huddling and a decrease in total activity relative to warm-acclimated group of mice only during the dark phase of 24 h period (Batchelder et al., 1983). Whether the activity pattern in huddling animals is affected by longer period of acclimation remains unexplored. To the extent of our knowledge, direct evaluation for maximum capacity for non-shivering thermogenesis (NSTmax) has not been examined in small mammals living in group during adaptation to low temperature. Few studies only mentioned that mice living in group developed less brown adipose tissue in the cold (Heldmaier, 1975) or the activity of uncoupling protein 1 was suppressed due to increased housing mice density (Himms-Hagen and Villemure, 1992).

Brandt's voles (*Lasiopodomys brandtii*) are small non-hibernating herbivorous rodents that are widely distributed in the dry steppe zone of Mongolia, the southeast of Baikal region of Russia and the Inner Mongolian grasslands of Northern China (Zhang and Wang, 1998; Avirmed, 2003). Their habitat is characterized by extreme continental climatic condition with long cold and dry winters and deep frozen soil. Brandt's voles live in family groups in complex burrow systems. The physiological mechanisms of individual Brandt's vole to the cold acclimation have been studied (Li and Wang, 2005; Zhang and Wang, 2006; Tang et al., 2009; Zhang et al., 2015). For example, individual Brandt's voles exposed to cold increase their resting metabolic rate (RMR), energy intake and uncoupling protein content in brown fat (Zhang and Wang, 2006). To date, there is no information on their adaptive strategies when they are in groups. Therefore, our study aimed to determine the significance of huddling behavior in group of Brandt's voles on energy conservation, NSTmax, T_b_ regulation, activity pattern, and body composition as a function of T_a._ We predicted that (i) huddling would reduce energy intake, (ii) change body composition, (iii) reduce energy expenditure, (iv) reduce NSTmax, (v) keep T_b_ higher, (vi) and reduce activity in comparison to separated voles in the cold.

## Materials and methods

### Experimental design and animals

Adult female voles with a body mass of 28–70 g and about 4 months old from a breeding colony at Institute of Zoology, CAS were examined. They were housed under laboratory condition with light regime 16L: 8D h s (light on from 4:00 to 20:00) and T_a_ 23 ± 1°C. For experiments we preferred to use 4 sibling voles, but also substituted the lacking siblings by up to 2 voles of similar age; animals had 3 weeks to acclimate to the cage (42 × 27 × 20 cm) and cage mates. Individual voles were dyed for identification. The cages had 4 equal compartments separated by stainless steel walls with small holes (6 mm) and connected by passageways. For huddling groups the passageways were opened in order to provide free movement of voles; passageways were closed for separated groups, but they had possibilities of olfactory, visual and vocal contacts. To reveal the effects of huddling we compared 4 experimental groups in different grouping conditions (huddling and separated) and T_a_s (cold 4 ± 1°C and cool 23 ± 1°C) for 4 weeks of the experimental period. The group size (4 voles in each cage) used in this experiment was chosen to ensure compact groups in which most animals remained inactive in a huddle. There were 8 cages for Cool-H, 9 cages for Cool-S, 7 cages for Cold-H and 7 cages for Cold-S groups, respectively. In the experiment we used 31 cages with a total of 124 voles. During the experimental period voles were provided with standard rabbit pellet chow (Beijing KeAo Bioscience Co.) and water *ad libitum* and wood shavings as bedding except period of food intake measurements. The study was carried out in accordance with recommendations of the Animal Care and Use Committee of the Institute of Zoology, the Chinese Academy of Sciences. The protocol was approved by the Animal Care and Use Committee of the Institute of Zoology, Chinese Academy of Sciences (IOZ16042).

### Body mass and food intake

Body mass of voles was measured before experiment and once in every week during the experiment by using an electronic balance (Sartorius Model BL 1500, ± 0.1 g). Food intake was measured 3 times by groups, once before the experiment as baseline when all cages were in the same T_a_ (23°C) and huddled together and next 2 measurements in the second and third week during the treatment. Each food intake measurement lasted for 3 consecutive days and animals were provided with *ad libitum* food and 5 g tissue paper in group voles and 3 g tissue paper for each separated voles for absorbing urine. The food given to the voles was weighed and dry mass calculated according to the measured water content of samples. Food intake was calculated by subtracting the uneaten from the total offered food. Energy contents of the food and feces were determined with a Parr 1281 oxygen bomb calorimeter (Parr Instrument Company, Moline, Illinois, USA). Gross energy intake (GEI), digestible energy intake (DEI) and apparent digestibility were calculated based on (Grodzinski and Wunder, 1975; Liu et al., 2003):

$$\begin{array}{lll} \text{GEI }\left(\text{kJ}/\text{day}\right)\; & = & \text{ Food intake }\left(\text{g}/\text{day}\right)\\ \times \text{ energy content of food }\left(\text{kJ}/\text{g}\right)\\ \text{DEI }\left(\text{kJ}/\text{day}\right)\; & = & \text{ GEI}-\text{dry mass of feces }\left(\text{g}/\text{day}\right)\\ \times \text{ energy content of feces }\left(\text{kJ}/\text{g}\right)\\ \text{Digestibility }\left(\text{\%}\right)\; & = & \;\left(\text{DEI}/\text{GEI}\right)\;\times \;100\%. \end{array}$$

### RMR and NSTmax

RMR was measured by groups of voles both huddling (4 voles in a transparent plastic chamber with a volume of 5.8L) and separated (4 voles in the same chamber but separated by double-layered dividing meshes) for 3 h at their acclimation T_a_s 4 and 23°C in open-circuit respirometry system (TSE labmaster, Germany). The air flow rate was 3L/min. The open circuitry respirometry system monitored 4 metabolic chambers in one running of the measurement. Three metabolic chambers were used for oxygen consumption of group voles and one blank chamber used for a baseline in sequence, but all sampled in every 6 min. We took the average of consecutive, stable and minimum 3 readings of oxygen consumption as the RMR at least after 1 h acclimation to the chamber.

The capacity for NST was determined individually at T_a_ 25°C 2 days after the RMR measurements. The volume of transparent plastic chamber was 2.7 L, and flow rate was 1 L/min. We took voles from their acclimation T_a_ room and injected norepinephrine (NE) subcutaneously (Shanghai Harvest Pharmaceutical Co. Ltd) to induce the maximum NST. The dosage of NE was calculated by the formula NE (mg/kg) = 2.53W^−0.4^ for Brandt's voles (Wang and Wang, 2006). The NSTmax was estimated as the stable 4 highest consecutive 3–min readings of oxygen consumption after 15–20 min of injection during 1 h measurements.

### Wet thermal conductance (C)

Group wet thermal conductance was determined by formula C = RMR/ (T_b_-T_a_) (Scholander et al., 1950; McNab, 1980). However, as we did not measure T_b_ directly during RMR, we assumed that the averaged daytime T_b_ derived from transponder data from each group at end of the acclimation period were equivalent to the T_b_ during RMR measurements because voles were exposed to same T_a_.

### Core body temperature (T_b_) and locomotor activity (activity)

T_b_ and activity were recorded via intraperitoneally implanted transponders (G2 E-Mitter, to ± 0.01°C, STARR life sciences). Prior to implantation surgery we calibrated the transponders in water bath to the nearest 0.1°C against a precision mercury thermometer. Before the surgery the transponder and surgical apparatus were sterilized in a 75% by volume alcohol solution for 30 min. Animals were anesthetized by injection of pentobarbital sodium (1%) with a dose of 50 mg/kg. After sterilizing the skin with an iodophor (Nanjing modern sanitation & anti-epidemic products Co.Ltd) we made a small (~1 cm) incision along the midline of the abdominal skin and muscles ~ 1 cm caudal to the diaphragm to open the abdomen. After insertion of the transponders, the wound was closed with absorbable PGA surgical suture (Jinhuan Model R413, 4/0) and sterilized with iodophor again. During surgery, a temperature controlled blanket (Temp control II 908100 by TSE systems) was used to prevent hypothermia until animal had recovered from anesthesia about 2 h after the injection. After surgery voles were kept in a cage in 23°C room individually for 2 days, then transferred to the original cage; 10 days were allowed for recovery before experiments began. For T_b_ and activity recordings, all receivers were connected to a computer using the VitalView software. Data of T_b_ and activity were collected at 6-min intervals throughout the experiment (Chi and Wang, 2011).

### Body composition

All voles were sacrificed with CO_2_ asphyxiation between 08:00 and 11:00 at end of the 4 weeks. We weighed (Mettler PE360 to 0.001 g) the wet masses of interscapular brown adipose tissue (iBAT), liver and retroperitoneal and epigonadal white adipose tissues (WAT), gonads and total gut mass with content. After removing the visceral organs and iBAT, the body mass including WAT of above organs was weighed, then dried in an oven until mass was constant and weighed again to determine body dry mass. Total body fat was extracted from the dried carcass with WAT by petroleum ether extraction in a Soxtec apparatus (Avanti 2055, Foss, Sweden).

### Statistics

We used the software SPSS 17.0 for statistical analyses. Body mass and food intake were converted to percentage in order to show their patterns during the experimental period. For the statistical analyses data on body mass were arcsine transformed and analyzed by two-way ANOVA (T_a_ and grouping condition) at each time point and repeated measures.

Group food intake was analyzed by two-way ANCOVA (temperature and grouping condition) with body mass as covariate and repeated measures of ANCOVA. RMR by a group of voles and NSTmax by individual were analyzed by two-way ANCOVA with body mass as covariate. Dry and wet carcass mass, retroperitoneal and epigonadal WATs, total body fat, iBAT, liver, gonad and gut mass with content were analyzed by two-way ANCOVA with body mass as covariate.

Data of core T_b_ and activity were averaged for each day as well as daytime and nighttime during the experimental period. For statistical analyses, we used only last 2 weeks of data when all voles' physiological acclimation was completed and became stable. Core T_b_, activity and wet thermal conductance were analyzed by two-way ANOVA; differences in T_b_ and activity between daytime and nighttime were determined by *t*-test. Pearson's correlation was carried out to examine the relationship between core T_b_ and activity by hourly means of last 4 days. Significant group differences were further evaluated using least significant difference *post hoc* tests. All values were expressed as mean ± SE and *P* < 0.05 was considered to be statistically significant.

## Results

### Body mass and food intake

**Figure 1:**
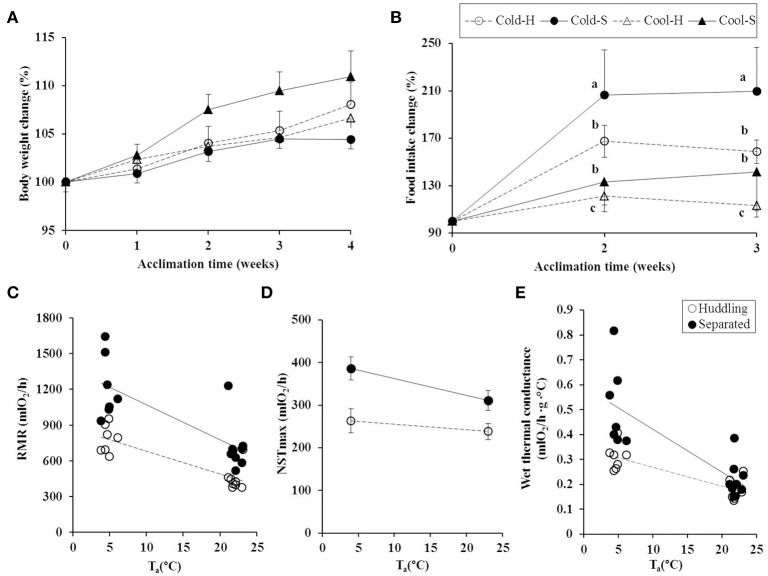
Body mass changes **(A)**, group food intake **(B)**, group RMR **(C)**, NSTmax **(D)**, and group wet thermal conductance **(E)** in huddling and separated Brandt's voles at cool and cold T_a_s. **(A)**: Cool-S, cool separated voles (*n* = 32); Cool-H, cool huddling voles (*n* = 28); Cold-S, cold separated voles (*n* = 25) and Cold-H, cold huddling voles (*n* = 27); **(B)**: Cool-S group (*n* = 8); Cool-H group (*n* = 7); Cold-S group (*n* = 6) and Cold-H group (*n* = 6); **(C)**: Cold-H group (*n* = 7); Cold-S group (*n* = 7); Cool-H group (*n* = 8) and Cool-S group (*n* = 9); **(D)**: Cold-H voles (*n* = 7); Cold-S voles (*n* = 7); Cool-H voles (*n* = 8); Cool-S voles (*n* = 9); **(E)**: Cold-H group (*n* = 7); Cold-S group (*n* = 7); Cool-H group (*n* = 8) and Cool-S group (*n* = 9). Values are means ± SE. Different small letters indicate significance (*P* < 0.05).

All groups increased their body mass [repeated measure, *F*_(4, 432)_ = 29.559, *P* < 0.001] during the course of experiment, but there was no difference among groups during the entire experiment [by temperature *F*_(1, 108)_ = 1.179, *P* = 0.28 and grouping condition *F*_(1, 108)_ = 0.278, *P* = 0.599] (Figure 1A).

Prior to experiment the voles from all groups were kept in same T_a_ (23°C) and could huddle with cagemates. Baseline food intake during this time did not differ among groups [*F*_(3, 22)_ = 0.275, *P* = 0.843]. After 2 weeks under experimental conditions the group food intake was significantly affected by T_a_ [*F*_(1, 22)_ = 148.180, *P* < 0.001] and grouping condition [*F*_(1, 22)_ = 67.892, *P* < 0.001]. The Cold-S groups showed the highest food intake in comparison to other groups, the Cold-H groups increased food intake to an intermediate level between the Cold-S and cool groups, but was not significantly different from Cool-S group (LSD *post hoc* test *P* = 0.509). Repeated measure analysis showed that cold groups increased food intake at the second measure and then kept it stable [Cold-S *F*_(2, 10)_ = 35.024, *P* < 0.001; Cold-H *F*_(2, 10)_ = 35.549, *P* < 0.001], while cool groups did not show any significant changes over time [Cool-S *F*_(2, 14)_ = 1.065, *P* = 0.371; Cool-H *F*_(2, 12)_ = 2.043, *P* = 0.172] (Figure 1B). When body mass adjusted food and gross energy intakes of different groups are considered, huddling groups significantly reduced food intake by 13.19 g/day (29%) and 7.61 g/day (26%), and also saved 156.99 kJ/day and 105.19 kJ/day digestible energy per day in cold and cool T_a_s at last measurement in comparison to separated groups, respectively (Table 1).

**Table 1:** Body mass adjusted food intake, gross energy intake, digestible energy intake, and digestibility in groups of Brandt's voles under different T_a_ and grouping conditions.

**Parameters**	**Cool-S (*n* = 8)**	**Cool-H (*n* = 7)**	**Cold-S (*n* = 6)**	**Cold-H (*n* = 6)**	**Saved energy at 23 °C**	**Saved energy at 4°C**
Food intake (g/day)	29.10 ± 1.83^b^	21.48 ± 1.98^c^	44.63 ± 1.94^a^	31.44 ± 1.94^b^	7.61	13.19
Gross energy intake (kJ/day)	489.70 ± 30.61^b^	364.47 ± 33.03^c^	759.17 ± 32.43^a^	535.33 ± 32.48^b^	125.23	223.84
Digestible energy intake (kJ/day)	359.91 ± 26.45^b^	254.72 ± 28.54^c^	528.24 ± 28.07^a^	371.25 ± 28.07^b^	105.19	156.99
Digestibility%	72.87 ± 1.1	70.0 ± 1.18	69.65 ± 1.16	69.44 ± 1.16	–	–

*Covariates appearing in the model are evaluated at the following values: BW = 203.7 g; BW, body weight; Cool-S, cool separated; Cool-H, cool huddling; Cold-S, Cold separated; Cold-H, Cold huddling. Different letters in each row indicate significant difference (P < 0.001)*.

### RMR and NSTmax

At the end of experiment the separated groups had higher group RMR (Cold-S: 1220.28 ± 99.79 ml O_2_/h; Cool-S: 714.66 ± 68.07 ml O_2_/h) than huddling groups (Cold-H: 784.33 ± 44.78 ml O_2_/h; Cool-H: 448.66 ± 36.48 ml O_2_/h) (grouping condition [*F*_(1, 26)_ = 22.07, *P* < 0.001]. The group RMR was increased by low T_a_ [*F*_(1, 26)_ = 37.347, *P* < 0.001], but there was no interaction between T_a_ and grouping condition on RMR [*F*_(1, 26)_ = 1.353, *P* = 0.255]. Huddling groups decreased RMR by 35.7% and 37.2% in cold and cool T_a_s, respectively (Figure 1C). The RMR of Cold-H group was intermediate between Cold-S and Cool-H groups (LSD *P* = 0.001), but were not significantly different from Cool-S group (LSD *P* = 0.433).

Separated voles had higher NSTmax (Cold-S: 386.48 ± 27.07 mlO_2_/h; Cool-S: 311.58 ± 23.40 mlO_2_/h) than huddling voles (Cold-H: 263.55 ± 28.55 mlO_2_/h; Cool-H: 238.06 ± 18.72 mlO_2_/h) (grouping condition [*F*_(1, 24)_ = 8.458, *P* = 0.008]. NSTmax was not changed by T_a_ [*F*_(1, 24)_ = 3.815, *P* = 0.063] or the interaction between T_a_ and grouping condition [*F*_(1, 24)_ = 0.928, *P* = 0.345]. NSTmax in huddling voles was significantly lower by 31% at cold and 23.5% at cool T_a_s (Figure 1D). Cold-H voles did not increase the NSTmax and maintained it at values similar to the voles in the cool.

### Wet thermal conductance (C)

Cold-exposed groups either huddling or separated had higher group wet thermal conductance than cool-exposed groups [*F*_(1.27)_ = 40.989, *P* < 0.001]. Huddling decreased group wet thermal conductance in cold T_a_ [*F*_(1.27)_ = 13.176, *P* = 0.001], while huddling in cool T_a_ did not change (Figure 1E). Cold exposure increased heat loss, but Cold-H groups were able to reduce it by 39% to an intermediate level between Cool-H and Cold-S.

### Core T_b_ and activity

**Figure 2:**
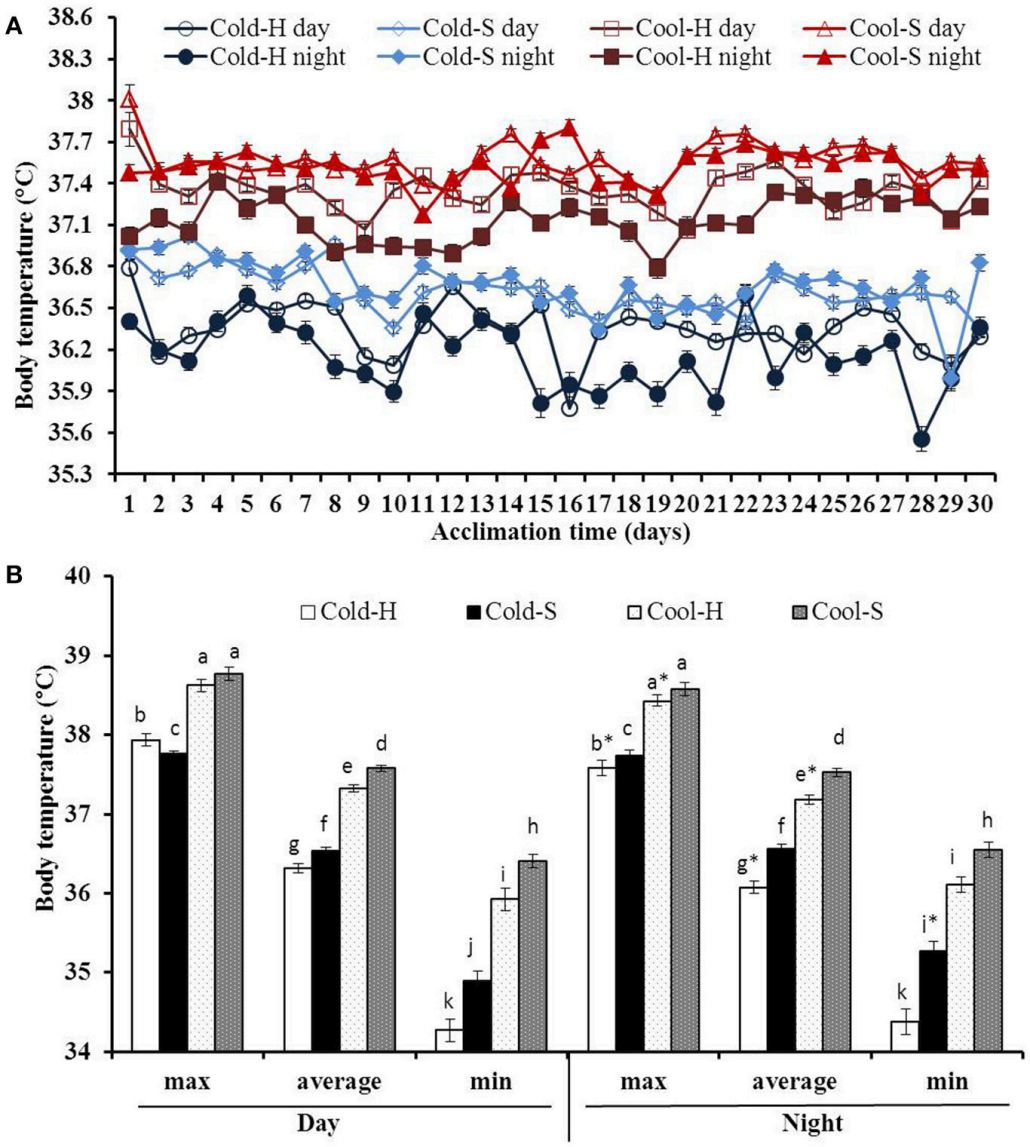
Daytime and nighttime average T_b_s during acclimation **(A)** and differences in maximum, average and minimum Tb **(B)** of voles from different experimental groups. Cool-H (*n* = 4); Cool-S (*n* = 5); Cold-H (*n* = 4); Cold-S (*n* = 4). Values are means ± SE. ^*^(star) indicates differences between daytime and nighttime values. Different small letters indicate the significant differences among experimental groups.

Both daytime and nighttime average T_b_s of voles fluctuated over the time of the experiment [*F*_(29.377)_ = 5.161, *P* < 0.001] and [*F*_(29.377)_ = 3.675, *P* < 0.001, respectively] (Figure 2A).

T_b_ was also significantly different among groups for daytime (Cool-S: 37.57 ± 0.04°C; Cool-H: 37.32 ± 0.05°C; Cold-S: 36.53 ± 0.04°C; Cold-H: 36.31 ± 0.06°C) [*F*_(3.55)_ = 349.908, *P* < 0.001] and nighttime (Cool-S: 37.52 ± 0.05°C; Cool-H: 37.18 ± 0.06°C; Cold-S: 36.56 ± 0.05°C; Cold-H: 36.06 ± 0.08°C) [*F*_(3.55)_ = 153.260, *P* < 0.001]. Huddling voles significantly decreased their nighttime average T_b_ by 0.14°C (in cool) and 0.25°C (in cold) in comparison to their daytime average T_b_ values (Cool-H: *t* = 2.547, *P* = 0.017, Cold-H: *t* = 3.292, *P* = 0.003) while separated voles did not show such differences (Figure 2B).

Maximum T_b_ of voles in the cool at daytime was higher than that of voles in the cold [*F*_(1.52)_ = 58.491, *P* < 0.001) with interaction between T_a_ and grouping condition [*F*_(1.52)_ = 11.844, *P* = 0.001]. At nighttime these values were significantly lowered in most groups except Cold-S voles. Minimum T_b_ was significantly increased only in Cold-S voles at night while there were no changes in other groups (Figure 2B).

**Figure 3:**
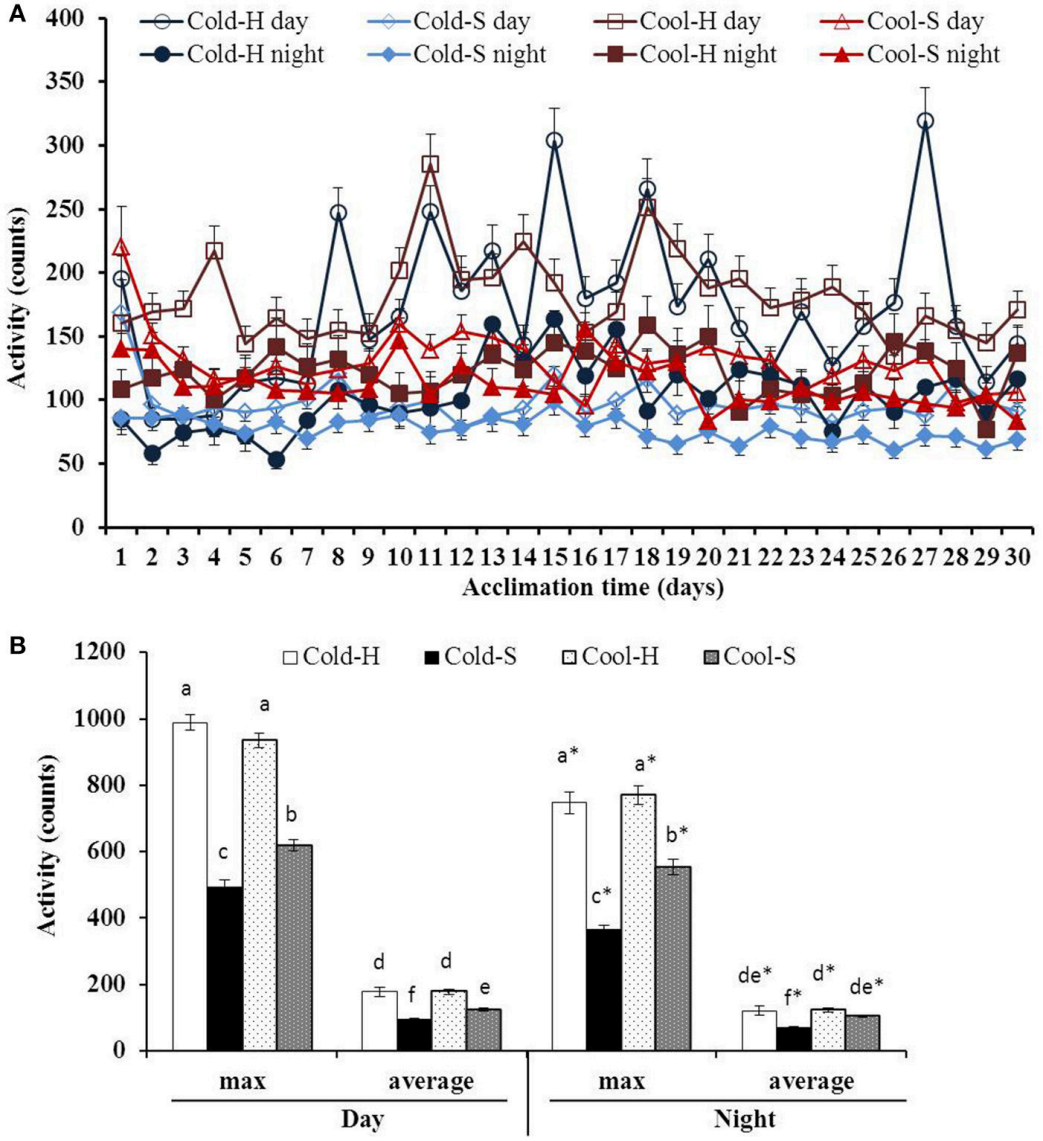
Daytime and nighttime activities during acclimation **(A)** and differences in maximum and average activity **(B)** of voles from different experimental groups. Different letters indicate significance among groups; ^*^(star) indicates differences between daytime and nighttime values.

Large fluctuations in daytime average activity were found especially in Cold-H and Cool-H voles with time [*F*_(29.377)_ = 5.714, *P* < 0.001] relative to nighttime average activity with time [*F*_(29.377)_ = 1.745, *P* < 0.05] (Figure 3A). Cold-H voles decreased their activity during the first week, then increased it to the level of Cool-H voles and had similar pattern until end of experiment (Mean difference = 0.71, *P* = 0.933) whereas Cold-S voles reduced activity until end of experiment.

The activity of huddling voles was higher than that of separated voles at both daytime [*F*_(1.52)_ = 59.912, *P* < 0.001] and nighttime [*F*_(1.52)_ = 20.192, *P* < 0.001]. Daytime average activity of voles in all groups was significantly higher than that of their nighttime activity (Cool-S: *t* = 3.610, *P* = 0.001; Cool-H: *t* = 5.571, *P* < 0.001; Cold-S: *t* = 8.048, *P* < 0.001; Cold-H: *t* = 2.844, *P* = 0.009) same as maximum activity (Figure 3B). Cold-H voles increased their activity by 1.7 fold in comparison to Cold-S voles. Pearson correlation on core T_b_ and activity was positive in all experimental groups (Cool-S: day *r* = 0.727, *P* < 0.001, night *r* = 0.866 *P* < 0.001; Cool-H: day *r* = 0.634, *P* < 0.001, night *r* = 0.882, *P* < 0.001; Cold-S: day *r* = 0.479, *P* < 0.001, night *r* = 0.518, *P* < 0.001; Cold-H: day *r* = 0.641, *P* < 0.001, night *r* = 0.457, *P* < 0.001).

### Body composition

Cold-exposed voles had higher iBAT mass than cool-exposed voles [*F*_(1, 57)_ = 6.611, *P* = 0.013] and separated voles had higher iBAT mass than huddling voles [*F*_(1, 57)_ = 5.836, *P* = 0.019]. iBAT mass of Cold-S voles was higher than in voles from other groups [*F*_(3, 61)_ = 6.810, *P* = 0.001]. Total gut wet mass of cold-exposed voles was significantly higher than that in cool exposed voles [*F*_(1, 57)_ = 47.443, *P* < 0.001], but there was no difference by grouping conditions [*F*_(1, 57)_ = 2.808, *P* = 0.099]. Wet liver mass of huddling voles was significantly lower than that in separated voles [*F*_(1, 57)_ = 9.674, *P* = 0.003] at cool T_a_, whereas such differences was not significant in cold groups. Total body fat of huddling voles was significantly lower than that in separated voles [*F*_(1.57)_ = 6.618, *P* = 0.013], but there was no significant difference between cold and cool groups [*F*_(1.57)_ = 2.689, *P* = 0.107]. Both wet and dry carcass masses of huddling voles were lower than that in separated voles [wet mass, *F*_(1, 57)_ = 8.479, *P* = 0.005; dry mass, *F*_(1, 57)_ = 7.795, *P* = 0.007] and cold exposure reduced carcass mass compared with cool condition [wet mass, *F*_(1, 57)_ = 19.327, *P* < 0.001 and dry mass, *F*_(1, 57)_ = 4.053, *P* = 0.049]. There were no differences in body water content, retroperitoneal and epigonadal WATs, and gonad masses among groups (Table 2).

**Table 2:** Effects of T_a_ and grouping condition on body composition of voles from different groups.

**Parameters**	**Cool-S (*n* = 20)**	**Cool-H (*n* = 16)**	**Cold-S (*n* = 13)**	**Cold-H (*n* = 13)**	**Effects**
Final body mass (g)	60.460 ± 2.984^a^	45.806 ± 2.38^c^	55.784 ± 3.828^ab^	50.115 ± 2.680^bc^	G^**^
Carcass wet mass (g)	44.749 ± 2.276^a^	32.502 ± 1.589^b^	39.273 ± 2.785^ac^	34.351 ± 2.013^bc^	T^***^,G^**^
Carcass dry mass (g)	26.194 ± 1.811^a^	16.097 ± 1.067^c^	21.927 ± 2.077^ab^	17.602 ± 1.489^bc^	T^*^,G^**^
Body water (g)	18.555 ± 0.702	16.4049 ± 0.75	17.3458 ± 0.870	16.7491 ± 0.633	ns
Retroperitoneal WAT (g)	2.1716 ± 0.283^a^	0.8131 ± 0.131^ab^	1.5878 ± 0.321^ab^	0.9962 ± 0.221^b^	ns
Epigonadal WAT (g)	1.5318 ± 0.236	0.5986 ± 0.122	1.1438 ± 0.204	0.7646 ± 0.181	ns
Total body fat (g)	17.9326 ± 1.593^a^	8.8749 ± 0.944^b^	14.043 ± 1.842^ab^	10.1962 ± 1.332^b^	G^*^
Interscapular BAT (g)	0.328 ± 0.0355^b^	0.1911 ± 0.022^b^	0.4199 ± 0.056^a^	0.2486 ± 0.026^b^	T^*^,G^*^
Liver wet mass (g)	2.7373 ± 0.251^a^	2.1691 ± 0.215^b^	2.506 ± 0.212^ab^	2.5272 ± 0.342^ab^	G^**^
Gonad wet mass (g)	0.1355 ± 0.011	0.0931 ± 0.012	0.1367 ± 0.011	0.1005 ± 0.013	ns
Total gut wet mass (g)	6.583 ± 0.350^b^	5.7635 ± 0.414^b^	8.1552 ± 0.456^a^	8.1592 ± 0.371^a^	T^***^

Values are expressed as means ± SE. Different letters in each row indicate significant difference (P < 0.05). T, temperature; G, grouping condition; ns, not significant;

* P < 0.05;

** P < 0.01;

*** *P < 0.001*.

## Discussion

Huddling is social thermoregulatory behavior important to the adaptation to low ambient temperature for reducing heat loss, energy expenditure and maintaining body temperature of animals to survive the cold (Bustamante et al., 2002; Gilbert et al., 2010). Our study shows that huddling substantially affects thermal energetics, thermal conductance, body composition, NSTmax and iBAT mass of voles and results in a reduction of T_b_ and an increase of activity. These effects of huddling were stronger at low T_a_.

### Changes in body mass, energy intake and body composition

The voles of all groups increased body mass during the experiment regardless of T_a_ or grouping condition. Cold exposure increased food intake of cold groups more than cool exposed groups to reduce energetic challenges as for individuals exposed to cold (Zhang and Wang, 2006). Huddling groups reduced food intake by 29% in cold and 26% in cool as compared with separated groups and their digestible energy savings reached to 156.99 and 105.19 kJ/day respectively supporting our prediction. Because the 23°C was below lower critical temperature of thermoneutral zone (TNZ) (27.5°C−32.5°C) of Brandt's voles (Li and Huang, 1994), the Cool-H voles increased their metabolism as the T_b_ and T_a_ differential increased. For instance, non-reproductive Mongolian gerbil individuals increased their energy intake with decreased T_a_ from 50.3 ± 3.7 kJ/day at 30°C (within TNZ 26–38°C) to 70.2 ± 3.9 kJ/day at 21°C and 109.3 ± 6.7 kJ/day at 10°C (Yang et al., 2013). While mice under standard conditions (21°C) display energy expenditure 3.1 times higher basal metabolism (Fischer et al., 2018). For the group housing at TNZ, only few studies focused on mice because of their biomedical research significance (Gordon et al., 1998, 2014; Maher et al., 2015). Both singly and group housed mice preferred relatively warm T_a_s ~ 29°C during the light phase in temperature gradient tests higher than T_a_ of 22°C in animal facilities (Gordon et al., 1998). Group of 3 mice housed in the thermocline (floor temperatures 23–39°C) remained at warm end and had significantly smaller livers and kidneys and increase in tail length compared to group of mice in the isothermal runaway (22°C) as well as the cage controls (22°C). But within their TNZ of 30°C the inclusion of cagemates did not influence most physiological parameters in mice (Maher et al., 2015). This is reasonable because both single and group housed mice have thermal comfort at their TNZ. Obviously, when T_a_ decreases, effect of huddling in energy conservation is apparent. Reduced food intake in huddling groups of Brandt's voles was consistent with previous studies in several small rodent species (Prychodko, 1958; Springer et al., 1981; Kauffman et al., 2003; Nuñez-Villegas et al., 2014) at low T_a_. Except for the reduction in iBAT, huddling voles also had lower carcass masses and total fat, but increased the gut masses. Therefore, the decrease in overall carcass mass of huddling voles may be associated with decrease of their metabolic rate. Moreover, huddling remodels gut microbiota to affect host's energy metabolism during cold exposure (Zhang et al., in press).

### Changes in RMR, NSTmax and wet thermal conductance

Endotherms living in cold environment maintain normothermic and energy balance through an activation of mechanisms that increase the heat production and conservation (Liu et al., 2009). The metabolic rate and NST for thermoregulation are increased during cold exposure in Brandt's voles (Li et al., 2001; Zhang and Wang, 2006), prairie voles (*Microtus ochrogaster*) (Wunder et al., 1977) and root voles (Wang et al., 2006). As expected, cold-exposed voles increased RMR. However, the group RMR of huddling groups was lower by 35.7% and 37.2% at 4°C and 23°C of T_a_, respectively than in separated groups. The reduction of RMR in huddling group of Brandt's voles was within the range 8–53% of reduction in studies of huddling in many other animals (Gilbert et al., 2010). Furthermore, we importantly found that NSTmax was not increased in Cold-H voles and remained at the same level as for voles from cool groups while it was significantly increased in Cold-S voles. The low NSTmax in Cold-H voles was relative to their lower iBAT masses. It has also been shown that a pair of mice exposed to cold developed less BAT mass than single mice (Heldmaier, 1975) and the thermogenic state of BAT mitochondria and the content of total uncoupling protein were reduced by number of mice in group (Himms-Hagen and Villemure, 1992). Through decrease in exposed body surface area and increase in local heating huddling voles do not need to increase thermogenesis and thus are able to reduce thermoregulatory costs.

With regard to heat loss at low T_a_ the rate of heat loss in Brandt's voles is increased in comparison to that at 23°C due to high thermal conductance and surface to volume ratio. The group wet thermal conductance of Cold-H group was lower than that of Cold-S group, but higher than in cool groups. Huddling groups reduced their group thermal conductance through reduced surface area and decreased their fur thickness in contact zones and thus transferred heat between each other with less loss to the environment. Interestingly, we observed that some separated voles (not huddling) molted and quickly refreshed the pelage at beginning of the experiment especially around the neck and interscapular area to improve their insulation at cold T_a_.

### Changes in core T_b_ and activity

Maintenance of high constant T_b_ is an important feature of mammals and birds. Endothermic animals expend great quantities of energy to regulate and maintain constant internal thermal conditions and functional processes over wide range of T_a_s (Bennett and Ruben, 1979). Surprisingly and to the contrary our prediction, Cold-H voles had the lowest daytime (36.31 ± 0.06°C) and nighttime (36.06 ± 0.08°C) T_b_s than that of other groups. To our knowledge, this decrease of core T_b_ in huddling Brandt's voles is the first observation especially among small adult homeothermic rodent species. A decrease of T_b_ has been recorded only in huddling birds. Greater snow goose goslings (*Chen caerulescens atlantica*) growing in arctic environments lowered T_b_ by 0.3 ± 0.5°C during huddling (Fortin et al., 2000), and Emperor penguin (*Aptenodytes forsteri*) decreased T_b_ by 0.9°C during huddling in the Antarctic winter (Gilbert et al., 2007). In contrast, T_b_ was increased in huddling deer mice (*Peromyscus maniculatus*) (Andrews and Belknap, 1986) and townsend voles (*Microtus townsendii*) (Andrews et al., 1987). Moreover, a recent study on hamsters (*Phodopus sungorus*) showed that radiant heat exposure by heat lamp (mimic to basking) at low T_a_ reduced core T_b_, metabolic rate and thermal conductance (Geiser et al., 2016). Similarly in our study, huddling decreased T_b_ and RMR in Brandt's voles in the cold. This T_b_ decrease is probably explained by the fact that inside huddle bout the voles relaxed endothermic thermoregulation and maintenance of high normothermic T_b_ setpoint. Thus, the decrease of T_b_ contributed to reduction of heat loss by decrease in differential between T_b_ and T_a_. It is likely linked to the heat exchange among huddling voles, local surrounding heating (Hayes et al., 1992; Gilbert et al., 2012) and increased local surface T_b_ at contact zones between animals (Nuñez-Villegas et al., 2014; Bautista et al., 2017).

Interestingly, the activity of Cold-H voles was higher in comparison to Cold-S voles and opposite to our prediction. As Brandt's voles are diurnal animal, daytime T_b_ and activity of Cold-H voles were higher than at nighttime. Therefore, it seems that this species does not need to reduce the activity to save energy, but keep the activity same as Cool-H voles. Thus, energetic advantages achieved by reduction in RMR, NSTmax and T_b_ during huddling allowed Cold-H voles to increase their activity. However, T_b_ and activity were correlated in all groups in present study. The explanation that high activity is to increase T_b_ is not supported because the huddling voles had higher activity, but lower T_b_. The study involving 8 species showed that the temperature rhythm is not a byproduct of the activity rhythm, because T_b_ during the active phase of the daily cycle was higher than T_b_ during the inactive phase irrespective of the activity level prevailing during each phase (Refinetti, 1999). Gebczynski and Taylor (2004) have reported that NST plays more important role than activity in shaping of circadian rhythm of T_b_. In our study Cold-H voles did not increase the mass of iBAT, but rather increased their activity significantly by 1.7 fold than in Cold-S voles. Girardier et al. (1995) showed that obese rats with atrophied BAT and lean rats with active BAT increased their moving distance by 3.4 fold and 1.4 fold at cold exposure, respectively. Thus activity-associated thermogenesis was the predominant thermogenic source for obese rats whereas such a correlation was not found in lean rats. In addition, balancing the activity-induced increase in T_b_ with lower T_b_ minima allows exercised animals in cold environments to benefit from both maintained activity and any energy savings afforded by lower T_b_s (Glanville and Seebacher, 2010). Moreover, in the wild the Brandt's voles huddling in winter chamber need to move for feeding to a storage chambers located on the periphery of burrow system. The increased activity in huddling voles may also be related to disturbance of other voles in order to occupy a better position in a huddle.

## Conclusion

Our study shows that huddling in Brandt's voles is an important cooperative behavior to reduce metabolic cost for thermoregulation. The pronounced effects of huddling are achieved not only by a decrease in energetics but also involved decreases in NSTmax and iBAT mass, and importantly a decrease of core T_b._ The energetic benefits of huddling are likely more extensive than the cost for the increase of activity. Therefore, Brandt's voles can remain active for a longer period and maintain their body condition without increased energetic costs during cold exposure. This study highlights the ecological significance of huddling behavior for maintenance of individual fitness at low costs, and thus survival of population during severe winter in small mammals.

## Author contributions

GS, X-YZ, and D-HW conceived and designed the study. GS and Q-SC conducted the experiment. GS, X-YZ, and Q-SC analyzed the data. GS drafted the manuscript which discussed and improved by X-YZ and D-HW. All authors read and approved the final manuscript.

### Conflict of interest statement

The authors declare that the research was conducted in the absence of any commercial or financial relationships that could be construed as a potential conflict of interest.
